# A comprehensive analysis on child mortality and its determinants in Bangladesh using frailty models

**DOI:** 10.1186/s13690-017-0224-6

**Published:** 2017-09-07

**Authors:** Jahidur Rahman Khan, Nabil Awan

**Affiliations:** Institute of Statistical Research and Training (ISRT), University of Dhaka, Dhaka, 1000 Bangladesh

**Keywords:** Trends, Determinants, Child mortality, Random effect, Bangladesh

## Abstract

**Background:**

Bangladesh has experienced a significant reduction of child mortality over the past decades which helped achieve the Millennium Development Goal 4 (MDG 4) target. But the mortality among under-5 aged children is still relatively high and it needs a substantial effort to achieve the Sustainable Development Goal (SDG) target and decelerate the current rate of under-5 mortality. At this stage, it is hence important to explore the trend and determinants of under-5 mortality in order to reduce the vulnerability of child’s survival. The aim of this study is to explore the trends and identify the factors associated with mortality in children aged less than 5 years in Bangladesh.

**Methods:**

Data from three repeatedly cross-sectional Bangladesh Demographic and Health Surveys (BDHSs) for the year 2007, 2011 and 2014 were used. A stratified two-stage sampling method was used to collect information on child and maternal health in these surveys. Cox’s proportional hazards models with community and mother level random effects (or frailty models) were fitted to identify the associated factors with under-five mortality.

**Results:**

Our study reveals that urban-rural disparity in child mortality has decreased over the time. The frailty models revealed that the combined effect of birth order and preceding birth interval length, sex of the child, maternal age at birth, mother’s working status, parental education were the important determinants associated with risk of child mortality. The risk of mortality also varied across divisions with Sylhet division being the most vulnerable one. Moreover, significant and sizable frailty effects were found which indicates that the estimations of the unmeasured and unobserved mother and community level factors on the risk of death were substantively important.

**Conclusion:**

Our study suggests that community-based educational programs and public health interventions focused on birth spacing may turn out to be the most effective. Moreover, unobserved community and familial effects need to be considered along with significant programmable determinants while planning for the child survival program.

## Background

Child mortality is a vital indicator of child health and overall national development [1]. According to World Health Organization (WHO) [2], a substantial global progress has been made in reducing child deaths, from 12.7 million in 1990 to 5.9 million in 2015. Since 1990, the global under-5 mortality rate has dropped 53%, from 91 deaths per 1000 live births in 1990 to 43 in 2015. The world as a whole has been accelerating progress in reducing the under-5 mortality rate. Between 1990 and 2015, 62 of the 195 countries with available estimates met the Millennium Development Goal 4 (MDG 4) target of a two-thirds reduction in the under-5 mortality rate. Among them, 24 are low- and lower-middle income countries. Currently, 79 countries have an under-5 mortality rate higher than 25 deaths per 1000 live births [2].

Among these countries, Bangladesh has also registered a substantive acceleration. Bangladesh has experienced a remarkable change in child mortality rates over the few decades, from 133 deaths to 46 deaths per 1000 live births with a rate of 65% decline in the period 1989-2014 [3]. Over these decades, extensive changes have occurred in health policy related to maternal health and newborn care, which could be the underlying reason of reduced child mortality [4]. Moreover, a notable increase in the coverage of interventions relevant to child survival, such as births in a health facility, skilled birth attendance, antenatal care visit, coverage of breastfeeding within 1 h of birth and exclusive breastfeeding for children etc. had a significant contribution to reducing child mortality [4]. But the reduction in infant and newborn mortality has happened more slowly with a rate of 56 and 46% in the period 1989-14, respectively [5]. Although child mortality rate is decreasing over time, Bangladesh has to further reduce child mortality to obtain the Sustainable Development Goals (SDGs) [3]. Moreover, about 20% further reduction in infant mortality is needed to achieve the Health, Population, and Nutrition Sector Development Program (HPNSDP) target of 31 deaths per 1000 live births in 2016 [6]. So, the study on child mortality is an important public health issue for Bangladesh. With the growing emphasis on the implementation of family planning programs in recent time, finding out the determinants of child mortality and its trends is also getting important [7].

The high fertility rate is boosted up by high infant and child mortality, because of the fear of death of the children at an early age. There are many factors which are closely related to the mortality experience among children such as maternal education, household income status etc [8–10]. Generally, non-educated mothers have more child deaths than others [11]. Some demographic variables are associated with infant and child mortality such as maternal age at marriage and during child’s birth, birth spacing pattern, parity, maternal height and weight, and size of the children at birth [8–10, 12]. Children of very young mothers (less than 20 years or 20-24 years) are at greater risk of death and rate of mortality is higher for older women (greater than 35 years, especially higher for women older than 40 years) [13, 14]. Many studies show that infant and child mortality is high among the first born, but relatively low among second and the third order births [15]. The length of the birth interval has a negative association with the infant and child mortality, i.e., the smaller the birth interval, the higher is the child mortality [12]. Many other factors such as immunization status of children and delivery practice may also influence infant and child mortality [16–18].

Some studies have been done by considering the necessity of analyzing the infant-child mortality and its determinants in Bangladesh [10, 19]. Kabir et al. [10] and the most recent study by Abir et al. [19] were attempted to identify important factors influencing infant and child mortality. The analyses of under-5 child mortality data in previous studies were conducted by means of simple Cox’s proportional hazards model considering time-to-event (death) data independent [10, 19].

An assessment of determinants of child survival is necessary to reduce child mortality rates [20]. This study examines, in particular, the socioeconomic and demographic factors associated with mortality among children in Bangladesh, and the extent to which the survival outcomes of children and siblings are associated. After accounting for different known determinants of child mortality this type of association of child survival outcomes has been attributed due to unobserved heterogeneity [21–23]. Moreover, this association of child survival outcomes arises in the context of clustered data. In developing countries, different studies of under-5 mortality have largely utilized data from Demographic and Health Surveys (DHS). This national representative survey suggests that the lifetimes of children from the same cluster are correlated, so are their risks of death, due to the sampling design. This kind of dependency is frequently found at family (mother) level or community level. From the methodological point of view, statistical models that ignore this type of clustering can make the study results biased because they violate the assumption of independence of event times. In this respect, proportional hazards models with random effect or frailty models are important because they allow for the correlation in survival experiences of children as well as siblings and expected to give accurate estimates of determinants of mortality. Frailty models are also important in estimating the effect of unmeasured and unobserved factors on the likelihood of death. In this study, we control for correlation between event times at the mother level and community level, which also helps to capture the effect of unobserved factors on the risk of child death.

Although there have been quite a few studies in the past identifying the socioeconomic determinants of child mortality in Bangladesh, due to the continuous interventions by the government and non-government organizations throughout the past few decades, we believe many of the health-related and societal aspects have changed and it is worthwhile checking back if the determinants have changed over the years or not. The objective of the present study is to assess the trends, socioeconomic and demographic determinants causing the death of children under five year of age in Bangladesh, which would help the policy makers take necessary measures to hasten the mortality decline.

## Methods

### Sampling design and variables

Our study is based on the most recent nationally representative Bangladesh Demographic and Health Surveys (BDHS): 2007, 2011 and 2014 [24]. Information from these surveys were collected at the individual level (ever-married women at reproductive ages), and at the community level. These repeatedly cross sectional surveys were designed to collect detailed information on a wide range of indicators such as fertility, marriage, family planning, mortality, breastfeeding practices, nutritional status, maternal and child health, awareness and behavior regarding HIV/AIDS etc. In BDHSs, a two-stage stratified sampling was used where 600 clusters (enumeration areas, EAs) were selected with on an average 30 households per cluster. All surveys were conducted in collaboration with National Institute of Population Research and Training (NIPORT), ICF International, USA, and Mitra & Associates. These three nationally representative surveys gathered information from a total of 10996, 17749, and 17863 households in the year 2007, 2011, and 2014, respectively.

In this study, we considered those children who were born within preceding five years from the survey years. Among the children, those who died before reaching their 5th birthday were treated as failure cases and children who were still alive and did not reach their 5th birthday were treated as censored cases during the analysis. We excluded non-original resident and twin data from this dataset. Covariates in our analysis consists of the maternal age at marriage (“ <18 years”, “ ≥18 years”), child’s sex (“male”, “female”), birth order (“1”, “2-4”, “ ≥5”), preceding birth interval (“first”, “short (<24 months)”, “medium (25–48 months)”, “large (≥49 months)”), maternal age at birth (“ <25 years”, “25–34 years”, “ ≥35 years”), parental educational level (“no education”, “primary”, “secondary or higher”), religion (“Muslim”, “others”), wealth index (“poor”, “middle”, “rich”), exposure to media (“yes”, “no”), maternal malnutrition (“underweight”, “normal”, “overweight or obese”), maternal working status (“yes”, “no”), paternal age (“ ≤25 years”, “26–35 years”, “ >35 years”), place of residence (“urban”, “rural”), division (“Barisal”, “Chittagong”, “Dhaka”, “Khulna”, “Rajshahi”, “Sylhet”) and survey year (“2007”, ‘2011”, “2014”). We have merged Rangpur and Rajshahi divisions as Rajshahi division for BDHS 2011 and 2014 to match with BDHS 2007. Exposure to media of mothers was categorized as: ‘yes’ if the respondent was either watching TV or listening radio or reading newspaper at least once a week, and ‘no’ if otherwise. Body mass index (BMI) was the key indicator of maternal nutritional status. We categorized BMI (kg/m^2^) as “underweight (BMI <18.5)”, “normal (18.5-24.99)”, “overweight or obese (≥ 25)”. To study the trends and determinants of child mortality in Bangladesh, we pooled the three cross-sectional survey data.

### Models

The duration of survival since birth in months was used in measuring the risk of death in childhood which was a time-to-event data. There were several possible model options. An event history analysis procedure which was proposed by Cox is usually used to examine the impact of various factors on the risk of death [25]. The main advantage of this model is that it accounts for the problem of censoring in data. Standard Cox’s proportional hazards model is applicable when time-to-event data are independent, but in this study, data are obtained from a cluster survey and assumed to be correlated. It is assumed that the correlations are due to unobserved cluster (community or mother) specific covariates. One approach is to adjust unobserved covariates known as frailty (random effect) in the standard Cox’s proportional hazards model which is popularly called a frailty model. The frailty model assumes that the risk of death of an individual is a function of measured factors and a random term on the baseline hazard due to the unobserved cluster effect. The model is of the form,

1 $$ h_{ij}\left(t\mid X_{ij}, u_{i}\right)=u_{i} h_{0}(t) e^{\beta'X_{ij}},  $$

for time-to-event data, where *i* (1,…,*n*) denotes the cluster, while *j* (1,…,*n*_*i*_) denotes the observation (child) within the cluster. The frailty, *u*_*i*_ is a random positive quantity shared within the groups. Here, *h*_*ij*_(*t*∣*X*_*ij*_,*u*_*i*_) is the hazard of child death at time *t*; *h*_0_(*t*) is the baseline hazard, *X*_*ij*_ is a vector of covariates with associated vector of fixed parameters *β*. The parameters of this model are estimated by maximizing the partial likelihood with respect to the parameters *β*. Different distributions can be considered for this frailty such as Gamma, lognormal, Gompertz etc. We assumed Gamma distributions for both the frailties corresponding to community and mother in this study. Frailty distribution is considered based on mathematical convenience. If an estimate of variance parameter significantly differs from zero this will indicate that unmeasured and unobserved factors shared by children of the same family or cluster have an impact on the risk of death, that means their survival risks are correlated. On the other hand, child mortality does not differ between communities or mothers if the variance estimate is zero. In frailty models, the likelihood of death depends on the measured factors and the unmeasured community or mothers effect and resulting hazard ratios are therefore mother or community specific which measure the effect of a particular variable on the risk of death within a particular mother or community. To explore the dependence in frailty models, Kendall’s *τ* is used which denotes the correlation of subjects’ outcomes within groups or clusters [26]. A closed-form expression exists for Kendall’s *τ* under the Gamma frailty model. In addition, to quantify the magnitude of the effect of clustering within clusters median hazard ratio (MHR) is used, which is the median relative change in the hazard of the occurrence of the outcome when comparing identical subjects from two randomly selected different clusters that are ordered by risk [27].

The hazard ratios (HR) and their 95% confidence intervals (CI) obtained from the Cox’s proportional hazards models with and without random effect were used to measure the associations of predictor variables with the under-5 deaths, which are the study outcomes in our case.

## Results

### Descriptive statistics

Table 1 presents the distribution of child survival status by mother’s age at birth and birth order over the years. Results show that about 7% of the first children died before reaching the fifth birthday among the mother with age at birth less than 25, which gradually decreased over the years (about 4.5% in 2014). For children with birth order 4–5, the percentage of child death reached from about 5% (in 2007) to about 4% (in 2014). For children with birth order higher than 5, the death toll was significantly high in 2007 which has decreased to a great extent later. We will be cautious to draw any conclusion from this piece of information though because the number of children with higher than 5 birth order was understandably low for mothers aged below 25. Among the mothers who aged 25–34 years during the birth of their children, the percentage of child death didn’t decrease much over the years for the first children. However, for birth order 4–5, the percentage of child death was about 3.5% in 2007 and 3% in 2014. The percentage of death also decreased slowly for birth order more than five and mothers aged above 35 years at birth, although this trend of declining over time is only slight while comparing with the same birth orders for the <25 and 25–34 years aged (at birth) mothers.

**Table 1:** Child survival and mortality by maternal age at birth and birth order (survey year wise stratified)

Variables		Birth order			*p*–value
Maternal age at birth (years) and Survey year	Status	1	4–5	5+	
<25 and 2007	Alive	1496	1633	33	<0.001
	Death	112	84	9	
	% Death	6.97	4.89	21.43	
<25, and 2011	Alive	2287	2398	35	0.5801
	Death	115	118	3	
	% Death	4.79	4.69	7.89	
<25, and 2014	Alive	2344	1913	21	0.3119
	Death	110	73	1	
	% Death	4.48	3.68	4.55	
25–34, and 2007	Alive	94	1092	488	0.246
	Death	4	40	27	
	% Death	4.08	3.53	5.24	
25–34, and 2011	Alive	161	1685	466	<0.05
	Death	5	44	25	
	% Death	3.01	2.54	5.09	
25–34, and 2014	Alive	175	1702	366	0.3377
	Death	7	51	16	
	% Death	3.85	2.91	4.19	
≥35, and 2007	Alive	6	89	221	0.8446
	Death	0	4	13	
	% Death	0	4.3	5.56	
≥35, and 2011	Alive	8	132	218	0.8545
	Death	0	5	11	
	% Death	0	3.65	4.8	
≥35, and 2014	Alive	8	123	162	0.3917
	Death	1	5	8	
	% Death	11.11	3.91	4.71	

**Fig. 1:**
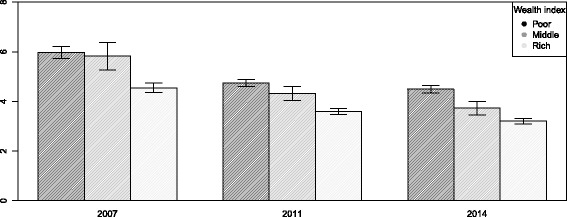
Trend of child mortality among the different groups of wealth index

**Fig. 2:**
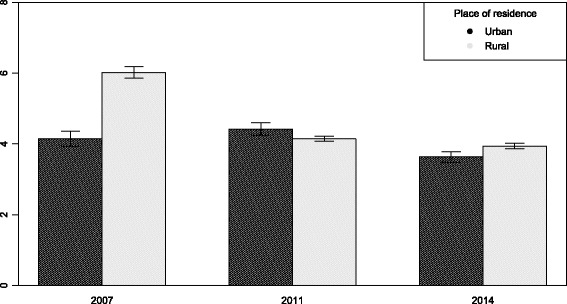
Trend of child mortality across the rural and urban areas of residence

There are notable disparities in child mortality across the household wealth status groups. The percentage of dead children from poor households were consistently more than the children from rich households. The graph shows that the percentage of mortality was declining among all groups, but the rate was slow in the period between 2011 to 2014 compared to the period between 2007 to 2011 (Fig. 1). The urban and rural differences in the prevalence of child mortality are highly notable in 2007, but over the time this difference has reduced. The rate of declining mortality was not much notable in urban areas over the time compared to the rural areas (Fig. 2).

### Risk factors of child deaths

Table 2 represents the potential risk factors associated with under-5 child mortality in Bangladesh. Cox’s proportional hazards model and frailty model were fitted to identify the socio-economic and demographic correlates of child mortality. The same set of covariates were used in all models. The selected socio-economic and demographic variables considered here in the model are mother’s age at marriage and at childbirth, sex of the children, parental education, place of residence, mother’s educational level, socio-economic status of household, preceding birth interval and birth order, religion, exposure to media, maternal malnutrition, mother’s working status, paternal age, division, and survey year.

The findings from this study revealed that the mother frailty model is the best model according to the likelihood ratio tests. The gender of the index child was a significant factor for childhood mortality. Female children were less likely to die within first five years of life compared to the male children (HR = 0.85, 95% CI = 0.75–0.98). The maternal age at birth was retained as a significant explanatory variable. Children from mothers with age at birth 25-34 years had a lower risk of dying compared with those born to mothers aged less than 25 years in all models. Similar results were found among the fathers with age 26–35 years (HR = 0.79, 95% CI = 0.64–0.97) compared to fathers with current age less than 26 years. The strong but unsurprising result is the effect of maternal and paternal education. The risk of mortality was significantly lower among children whose parents had secondary or higher education compared to the children whose parents had no education. For example, the risk was about 27 and 28% lower among children with secondary or higher educated mothers and fathers, respectively, compared to non-educated parents.

Preceding birth interval and birth order were also associated with mortality. In addition, children of 5 or higher birth order with a birth interval ≤24 months were at higher risk of mortality compared to the children who were the first births. But the probability of dying was declining significantly for the children with medium birth interval and 2 or higher birth order. The children from working women were 1.24 times at greater risk of dying than those who were not working. The likelihood of child mortality was 1.53 times higher among children from Sylhet division when compared with the Barisal division. The other divisions didn’t show much difference in child mortality. In addition, the probability of dying was significantly reduced over the time. The likelihood of child mortality was declined by 15 (in 2011) and 24% (in 2014) times compared to the year 2007. On the other hand, we did not find any significant association of maternal age at marriage, religion, household socioeconomic status, mothers’ exposure to media, and place of residence of the respondents with child mortality.

We also included two frailty terms that assumed to operate on a meaningful level. The mother frailty may capture any unobserved variables that operate on children born from the same mother, such as genetic factors and maternal competence. The community level frailty may account for the possible effects of climate, ritual practices or environmental factors within the community. The maximum log likelihood of the mother frailty model is -8151, which corresponds to the value 0.5734 of the estimated random effect variance, and a maximum log likelihood value of -8561 for the community frailty effect, which corresponds to the estimated random effect variance of 0.1066.

Median hazard ratio indicates that the median increase in the hazard of mortality when comparing the children at a community with higher mortality to the children at a community with lower mortality was about 37%, which was a whopping 119% (more than 2 times) when comparing the children of the mothers with higher and lower mortality. Kendall’s tau reveals that 5 and 22% of the variation in event times were due to variation between communities and mothers, respectively. According to likelihood ratio test, these parameters are highly significant and indicate that survival risks in childhood continue to vary due to unobserved factors in mother and community level. This implies that there are other factors which are affecting under-five mortality among children at mother and community level that are not explained by the observed covariates included in the model although the magnitude of most of the factors remains unchanged in the standard and frailty models. The results further suggest that unobservable factors related to the mothers were more likely to be associated with a higher risk of children dying before reaching the fifth birthday than the community level unobservables.

**Table 2:** Cox’s proportional hazards model and frailty model analysis: risk factors of child mortality in Bangladesh (BDHS, 2007–2014)

	Cox PH		Community frailty		Mother frailty	
Variable	HR	95% CI	HR	95% CI	HR	95% CI
Age at marriage (≥ 18 years)						
< 18 years	1.13	0.94–1.35	1.13	0.94–1.35	1.13	0.94–1.36
Religion (Muslim)						
Other	1.01	0.8–1.28	1.00	0.79–1.27	1.00	0.79–1.27
Sex of children (Male)						
Female	0.86^b^	0.75–0.98	0.86^b^	0.75–0.98	0.85^b^	0.75–0.98
Age at child birth (<25 years)						
25–34 years	0.82^c^	0.67–1.02	0.83^c^	0.67–1.02	0.83^c^	0.67–1.03
≥35 years	0.95	0.65–1.39	0.95	0.65–1.4	0.96	0.65–1.43
Maternal education (No education)						
Primary	0.87	0.73–1.05	0.87	0.73–1.05	0.87	0.72–1.06
Secondary or higher	0.73^a^	0.59–0.90	0.73^a^	0.59–0.90	0.73^a^	0.58–0.90
Paternal education (No education)						
Primary	0.96	0.81–1.14	0.96	0.81–1.14	0.96	0.81–1.14
Secondary or higher	0.73^a^	0.59–0.89	0.73^a^	0.59–0.89	0.72^a^	0.59–0.89
PBI and Birth order (First)						
Short and 2–4	1.18	0.94–1.48	1.16	0.93–1.46	1.05	0.84–1.33
Short and 5+	1.69^a^	1.16–2.46	1.67^a^	1.14–2.44	1.56^b^	1.06–2.31
Medium and 2–4	0.67^a^	0.54–0.82	0.67^a^	0.54–0.82	0.65^a^	0.53–0.80
Medium and 5+	0.55^a^	0.35–0.85	0.54^a^	0.35–0.84	0.53^a^	0.34–0.82
Large and 2–4	0.64^a^	0.51–0.79	0.64^a^	0.51–0.80	0.63^a^	0.50–0.79
Large and 5+	0.88	0.58–1.33	0.88	0.57–1.33	0.86	0.56–1.32
Wealth index (Poor)						
Middle	1.01	0.84–1.22	1.01	0.83–1.22	1.01	0.84–1.23
Rich	0.97	0.79–1.19	0.97	0.79–1.19	0.97	0.79–1.19
Exposure to media (No)						
Yes	1.00	0.85–1.17	1.01	0.86–1.18	1.00	0.85–1.18
Maternal malnutrition (Normal)						
Underweight	0.88^c^	0.75–1.02	0.87^c^	0.75–1.02	0.87^c^	0.74–1.02
Overweight or obese	1.08	0.86–1.35	1.08	0.87–1.36	1.09	0.86–1.37
Maternal working status (No)						
Yes	1.23^b^	1.05–1.45	1.24^b^	1.05–1.46	1.24^b^	1.05–1.47
Place of residence (Urban)						
Rural	1.01	0.86–1.19	1.01	0.86–1.2	1.01	0.86–1.19
Paternal age (≤25 years)						
26–35 years	0.78^b^	0.64–0.96	0.79^b^	0.64–0.96	0.79^b^	0.64–0.97
≥35 years	0.68^a^	0.53–0.88	0.68^a^	0.53–0.88	0.69^a^	0.53–0.89
Division (Barishal)						
Chittagong	1.04	0.8–1.34	1.03	0.79–1.34	1.03	0.79–1.34
Dhaka	0.92	0.71–1.2	0.92	0.7–1.2	0.92	0.7–1.21
Khulna	0.90	0.67–1.22	0.90	0.66–1.22	0.89	0.66–1.22
Rajshahi	1.01	0.78–1.30	1.00	0.77–1.30	1.00	0.78–1.30
Sylhet	1.53^a^	1.19–1.96	1.51^a^	1.17–1.96	1.53^a^	1.19–1.98
Survey year (2007)						
2011	0.85^b^	0.73–1.01	0.86^c^	0.72–1.01	0.85^c^	0.72–1.01
2014	0.75^a^	0.64–0.9	0.76^a^	0.63–0.90	0.76^a^	0.64–0.90
Variance of frailty		–		0.1066		0.5734
Kendall’s *τ*		–		0.0506		0.2228
MHR		–		1.3719		2.1907
Log–likelihood		-8652		-8561		-8151

*p*-value: a <0.01, b <0.05, c <0.10; PBI: Preceding birth interval

## Discussion

Over the last years, there has been a steady decline in the rates of under-5 mortality in Bangladesh which indicates the country’s level of improvement in the quality of life. These rates are also important in identifying the directions for the public health programs in Bangladesh [28]. The results of this study indicate that child’s death depends on gender, parent’s age at birth, parent’s education, preceding birth interval, mother’s working status. Among the divisions, Sylhet showed significantly higher child mortality than the others. A significant decline of the under-5 mortality was also observed over the years. High child mortality in Sylhet division was observed due to many factors, including religious influence, superstitions, and lower awareness about child and maternal health care [29]. Moreover, this division is also lagging behind the other divisions in terms of receipt of antenatal care, child delivery assisted by medically trained providers, and vaccination coverage among the children [29].

Significant mortality differentials were observed by maternal age at birth of the child. The findings reveal a higher risk of death for children of younger mothers which also confirm previous research findings [30, 31]. Maternal age at birth can influence child mortality through different perspectives. The higher risk of child death among younger mothers pertains because of immature reproductive systems and less stability to handle the complexities of childbirth [32]. Moreover, younger mothers are more likely to have low-birth-weight babies [33], which is associated with a higher risk of child death [34].

Mother’s education had a significant association with child survival, which contributes through different mechanisms. A high risk of child death among the illiterate mothers compared with the secondary or higher educated mothers is also consistent with other study findings [35, 36]. Educated mothers have better socioeconomic status, good knowledge on family health and childcare, are more conscious about child illness, preventive care and effective use of modern health services [35–37]. In addition, education also helps to change the traditional familial relationships regarding decision making and empowers the mothers in various issues like childcare which in turn plays a role in reducing child mortality [38, 39]. In contrast, maternal employment status turned out to be contributing negatively on child survival. Though apparently surprising, past studies have shown that maternal employment can have an adverse effect on the care of newborn, including infrequent breast feeding, and on personal care due to higher workload in performing the other traditionally ascribed roles within the family [40–42].

Our findings show that the risks of under-5 mortality were significantly higher for male children than for female children. The fact that girls have a biological advantage against many causes of death than boys can be a possible explanation of the higher risk of male child deaths [43–46], which is due to a lesser vulnerability to perinatal conditions, congenital anomalies, and infectious diseases [47].

Significant differences by the length of preceding birth interval were observed in this study. Findings from this study indicate that children with two or higher birth order who were born with shorter birth intervals (≤24 months) were at a greater risk of dying before five years of age, which is consistent with previous studies [18, 48–50]. A shorter length of the birth interval may negatively affect maternal health, increase the susceptibility of infectious diseases, and cause familial resource competition among children [48, 51]. Poor nutritional status, low birth weight, premature birth may influence the risk of having a small birth size for children resulting in a higher child mortality [52, 53].

This study has demonstrated that risk of child mortality varies due to unobserved factors not only at the family or mother level but also at the community level. The results of our frailty models suggest that the effects of unmeasured family and community level factors are likely to be important for child mortality in Bangladesh, specially the mother level frailty model was found particularly appropriate for the BDHS data. The family and community hetergeneity summarize the effects of various unobserved factors such biological, parental competence, genetic, behavioral, customs, maternal depletion, resource competition between siblings, cultural norms, environmental facts, quality of health facilities as well as care in health facilities and other unobserved factors.

## Conclusion

This study findings have important policy implications, especially in determining the program needs for a sustainable decline in child mortality rate, and in monitoring public health interventions. It is important to look beyond individual level and community level attributes. Increasing mother’s education and empowerment may help reduce childhood mortality. Reducing motherhood in younger ages and increasing the spacing between births are also necessary to reduce child deaths. Some other important contextual factors such as quality and care of health facility, cultural practices, customs, environmental condition etc. could not be addressed in this study due to unavailability of data in the DHS. The authors suggest further studies considering these unobserved factors that are likely to be associated with infant and child mortality to better understand the association between family and community level factors and child mortality in Bangladesh. Interventions and strategies should be targeted focusing on these characteristics to improve child health outcomes as well as future betterment of Bangladesh.

## Limitations

In this study, we have used cross-sectional data which limits any conclusions about the causality of the factors we have examined.
